# Preparation and Support Effect of Graphdiyne Nanotubes with Abundant Cu Quantum Dots

**DOI:** 10.3390/molecules29061410

**Published:** 2024-03-21

**Authors:** Yan Lv, Wenzhou Wang, Zhangwei Li, Fucang Liang

**Affiliations:** State Key Laboratory of Chemistry and Utilization of Carbon Based Energy Resources, College of Chemistry, Xinjiang University, Urumqi 830017, China

**Keywords:** graphdiyne nanotubes, Cu quantum dots, support, hydrogen generation

## Abstract

Graphdiyne (GDY) is considered a very attractive support for metal nanocatalysts due to its unique structure and superior properties. The metal–GDY interaction can significantly affect the performance of catalysts. Herein, GDY nanotubes abundant in in situ formed Cu quantum dots (QDs) (Cu-GDYNT) are prepared using the electrospun polyacrylonitrile nanofibers collected on the surface of electrolytic Cu foil as templates. The diameter of the Cu-GDYNT is controllable and the uniform size of the embedded Cu QDs is about 2.2 nm. And then, the uniformly dispersed and highly active supported catalysts of ruthenium nanoparticles (Ru_x_/Cu-GDYNT) are produced using the Cu-GDYNT as the support. Among them, the Ru_3_/Cu-GDYNT exhibit outstanding HER performance at all pH levels. Only 17, 67 and 83 mV overpotential is required to reach a current density of 10 mA cm^−2^ in 1.0 M KOH, 0.5 M H_2_SO_4_ and 1.0 M neutral PBS solutions, respectively. The sample exhibits 3000 CV cycle stability and 20 h continuous electrolysis without performance degradation in an alkaline medium. This work provides a new idea for constructing the GDY-supported metal nanocatalysts.

## 1. Introduction

Graphdiyne (GDY), as an increasing popular two-dimensional carbon material, possesses a sp- and sp^2^-hybridized carbon network and exhibits a unique structure as well as a series of superior properties [1,2,3]. Since it was first experimentally synthesized in 2010 by Li’s group in China, GDY has attracted intensive attention in the research fields of catalysis [4,5], energy conversion [6,7], membrane separator [8], energy storage [9,10], and so on. In particular, GDY exhibits striking properties in the construction of highly efficient heterogeneous catalysts. Abundant alkyne bonds in GDY can serve as homogeneous reaction sites to adsorb metal cations due to their strong electron-donating ability [11,12,13]. The strong d–π interaction between GDY and metal can promote the interfacial charge transfer and regulate the electronic structure of the active site [14,15]. The naturally porous structure resulting from the linking of the benzene ring and alkyne in GDY can expose more active sites and are beneficial for mass transport during the electrochemical reaction process [16,17]. The intrinsic charge carrier mobility of GDY (2 × 10^5^ cm^2^ V^−1^ higher than graphene and carbon nanotubes) endows the efficient electron transport to or from the metal center [18]. The high (electro)chemical stability of GDY ensures structural stability in the strong acid or base electrolyte [19,20]. In addition, the advantages of low-temperature growth on any substrate surface for GDY make the construction of efficient catalysts more attractive. Therefore, GDY is considered an ideal support for the in situ confinement growth of metal single-atom clusters, and nanoparticles, particularly. The favorable support effect and interface effect have been proven by numerous experiments and theoretical calculations [21,22]. Despite tremendous progress in the construction of GDY-supported metal nanocatalysts, it is still necessary to construct different kinds of GDY support, such as the change of aggregation form and the doping of non-carbon elements, to further increase its support effect, or provide other conveniences for the catalytic reaction.

The characteristic of GDY growing on any substrate surface suggests that we can use the template method to prepare GDY materials with different morphologies. In fact, such work has made some progress. GDY nanotube (GDYNT) arrays with a diameter of about 200 nm and a wall thickness of about 40 nm were prepared by Liu’s group using anodic aluminum oxide as a template [23]. The thickness of GDYNTs was changed to 15 nm after annealing. GDYNTs exhibit wall-thickness-dependent emission properties, demonstrating the potential of GDY for use in a new generation of electronic and optoelectronic devices. Chen’s group successfully prepared freestanding 3D GDY by adsorbing Cu nanoparticles and stabilizing them in the void of diatomaceous earth, as a catalyst and a template for the GDY coupling reaction [24]. The as-prepared 3D GDY displays a high specific surface area and porosity, showing great potential application in catalysts and energy storage. Huang’s group used copper oxide nanospheres as templates and catalysts to prepare hollow GDY nanospheres with controllable thickness and surface morphology [25]. Thanks to hierarchical nanostructures, a large specific surface area, and suitable cavities, the 3D hollow GDY nanospheres showed excellent performance as anode material. In addition, the GDY nanotubes [26], the 3D GDY with a hollow multishelled structure [27], and ordered GDY nanochannels [28] was prepared using the Cu nanowires, hollow multishelled Cu, and Cu substrate with nanowire arrays as a template and catalyst, respectively. However, the removal of the templates in these processes requires the use of either concentrated NaOH, HF acid, or the etching of FeCl_3_, which is time-consuming, costly, and can easily cause the loss of GDY yield and structural damage. Furthermore, the difficulty of adjusting the template size causes the uncontrollable structure of synthetic GDY, such as the diameter of GDY nanotubes.

Herein, we reported a facile strategy to produce GDY nanotubes with a controllable size using electrospun polyacrylonitrile (PAN) nanofibers as templates. The electrospun PAN nanofibers are controllable in size (by adjusting the spinning conditions) and easy to remove (dissolve in DMF at low temperature). More interestingly, the electrolytic copper foil used to collect PAN nanofibers is spontaneous split into Cu QDs and then embedded in the prepared GDYNTs in the process of catalyzing the coupling reaction. The Cu-GDYNT possesses a controlled structure, and the uniform size of the embedded Cu QDs is about 2.2 nm. Using the Cu-GDYNT as support, the supported catalysts of ruthenium nanoparticles (NPs) (Ru_x_/Cu-GDYNT) with a different Ru content are prepared without any surfactant. The as-prepared Ru_3_/Cu-GDYNT catalyst exhibits an excellent HER performance in the full pH range. This work provides a controllable preparation method of GDYNT and studies its support effects, but this idea can be applied to other GDYNT composites and applications in other fields.

## 2. Results and Discussion

### 2.1. The Preparation and Characterization of Cu-GDYNT

**Figure 1:**
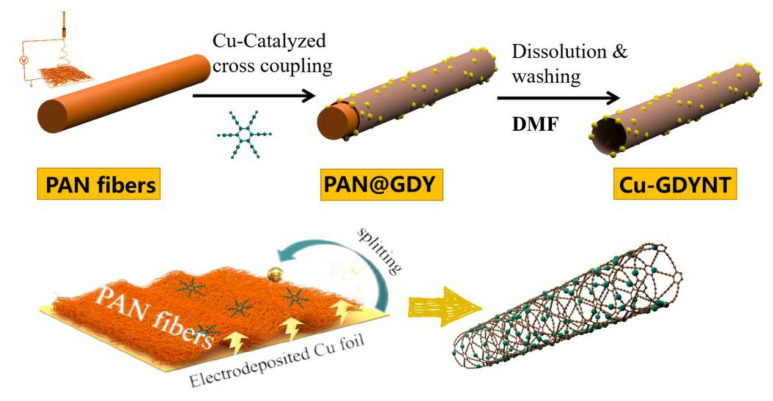
The fabrication process of the Cu-GDYNT.

Figure 1 schematically shows the preparation procedures for Cu-GDYNT (see the Experimental Section for details in the ESM). Electrospun PAN nanofibers using electrolytic Cu foil as the collector is prepared firstly. The parameters of the electrospinning process, including PAN concentration, stainless steel needle’s size, voltage, collecting distance, and propeller speed were set at 0.8 g mL^−1^, 0.9 mm (18G), 14 kV, 20 cm, and 0.08 rpm min^−1^, respectively. It is worth noting that the diameter of the PAN fiber can be adjusted by changing any of the above parameters, that is, it is easy to achieve template controllability [29]. Next, the GDY is controllably grown on the surface of the PAN nanofibers (PAN@GDY) via a modified Glaser–Hay coupling reaction as the reported previously [30]. The difference is that the electrolytic Cu foil (the collector of PAN fibers) and PAN nanofibers plays the roles of Cu-catalyst source and growth substrate of GDY, respectively. Compared with the general Cu foil (Sinopharm Chemical Reagent Co. Ltd., Shanghai, China, used as the catalyst for the synthesis of GDY in most reports [16,20,29]), the electrolytic Cu foil is composed of more grains (Figure S1), which can expose more crystal boundaries. In previous reports, Li and Zuo et al. have reported that the crystal boundary of Cu is the origin of its reaction activity, and the growth reaction of GDY can split the polycrystalline Cu into well-dispersed Cu QDs [31]. Therefore, it is expected that the GDY synthesized in this way should generate abundant Cu QDs on them. This will be confirmed by the subsequent characterization. Additionally, the thickness and surface morphology of GDY is controlled by the amount of monomer added. Finally, the PAN@GDY is put into *N,N*-dimethylformamide (DMF) at 50 °C to remove the PAN core and residual oligomer, and the GDY nanotubes with Cu QDs (Cu-GDYNT) are obtained readily. In fact, the use of DMF is necessary for the purification process of GDY, so we did not introduce additional reagents to remove the template. Due to the high solubility of PAN in DMF, this process is rapid and without ultrasound to peel, so the GDYNT with high yield and high quality can be obtained.

**Figure 2:**
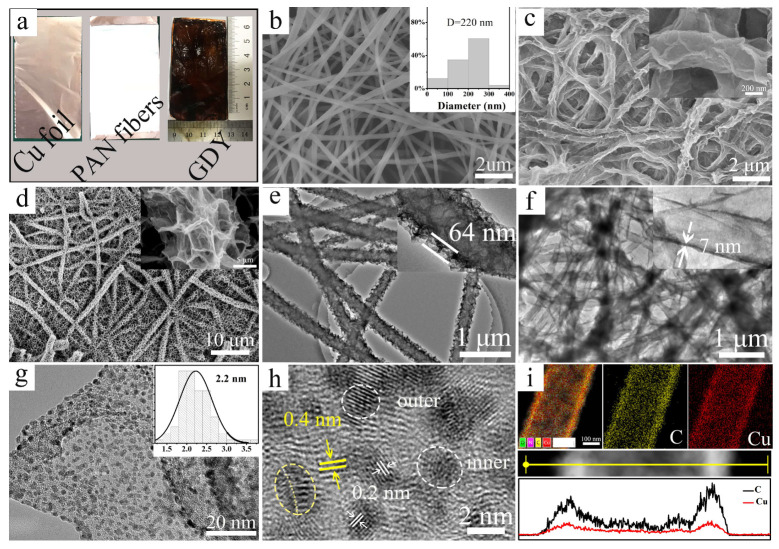
(**a**) Optical photographs of electrodeposited copper foil in the reaction process; the SEM images of (**b**) PAN nanofibers, the inset is a statistic diameter of PAN nanofibers; (**c**) Cu-GDYNT (20 mg HEB), (**d**) Cu-GDYNT (50 mg HEB); the TEM images of (**e**) Cu-GDYNT (50 mg HEB) and (**f**) Cu-GDYNT (20 mg HEB); (**g**,**h**) the high-resolution TEM images of Cu-GDYNT, the inset in (**g**) is the statistic diameter of Cu NPs; (**i**) the EDS-mapping and EDS-line scanning images (line scanning depends on atomic content).

Figure 2a shows the optical pictures of the surface of the electrolytic Cu foil in the reaction process. After GDY growth, the surface of PAN nanofibers uniformly and integrally changes from white to dark brown. Scanning electron microscopy (SEM) image (Figure 2b) shows that the PAN nanofibers of 220 nm are obtained by electrospinning. As a template, the diameter of the PAN nanofibers determines the inner diameter of the GDYNT. Therefore, the preparation of GDYNT with a controllable inner diameter is readily realized through the change in the electrospinning process. As shown in Figure S2, the GDYNT with a diameter of 80 nm is prepared using different PAN fibers (changing the stainless steel needle’s size from 0.9 to 0.6 mm) as a template.

Figure 2c,d show that the GDYNT retains the one-dimensional (1D) structure of the nanofibers, and makes the GDYNT cross-linked with each other in the form of chemical bonds at the intersection of the fibers, showing a 3D structure formed by the cross-linking of 1D nanofibers. This structure is beneficial to improve the mechanical strength and electrochemical response of the GDYNT. The difference between the morphology of Figure 2c,d lies in the different amounts of GDY monomer added during the reaction (HEB, 20 mg vs. 50 mg). It can be seen that cross-linked 2D GDY nanosheets grow on the surface of nanotubes with the increasing amounts of monomer, reflecting the two stages of GDY growth on the surface of PAN fibers [26]. First, GDY is tightly coated on the surface of PAN nanofibers to form GDYNT with many bumps on the surface (Figure 2c inset), and then these bumps are used as growth sites to grow GDY nanosheets vertically (Figure 2d inset). Compared to the smooth wall of carbon nanotubes, such a structure can provide a larger contact area for the reactants.

Figure 2e,f show the transmission electron microscopy (TEM) images of GDYNT (20 mg and 50 mg HEB). The hollow nanotube structure of GDYNT is observed directly. The inner diameter of GDYNT is about 220 nm, the axial length is more than 40 μm, and the wall thickness is about 7 nm uniformly at different positions. The formation of the uniform nanotube structure mainly depends on two reasons. On the one hand, PAN fibers prepared by electrostatic spinning have a uniform thickness and size. On the other hand, the electrodeposited Cu foil under the PAN fiber film, as the source of the catalyst for a coupling reaction, can ensure the uniform distribution of Cu ions on the fiber surface. The TEM images of GDYNT with a monomer content of 50 mg, show the same morphology features as SEM images. The height of the grown nanosheets is about 64 nm. Magnified TEM investigations (Figure 2g) confirm the speculation before that electrodeposited Cu foil is split into ultrafine Cu QDs and embedded in the plane of the GDY during the GDY growth process. A large number of Cu QDs with a size of about 2.2 nm uniformly dispersed on GDY sheets are observed. The images of different regions are shown in Figure S3, and display the same result. In the high-resolution TEM images (Figure 2h), the lattice fringe spacing of 0.20 nm belongs to the (111) crystal plane of the Cu. The layer spacing of GDY is increased to 0.4 nm due to the existence of Cu QDs, which facilitate the exposure of reaction sites, the immersion of reactants, and the overflow of generated gases [32]. Additionally, as shown in the yellow circles, the Cu QDs are in close contact with GDY, efficiently avoiding the aggregation of Cu QDs. EDX mapping images and compositional line profiles (Figure 2i) strongly substantiate the uniform spatial distributions of Cu elements in GDY nanosheets and confirm the hollow structure of GDYNT. Since there are abundant Cu QDs embedded in the GDYNT, the product is denoted as Cu-GDYNT below.

### 2.2. The Research on the Support Effect of Cu-GDYNT

**Figure 3:**
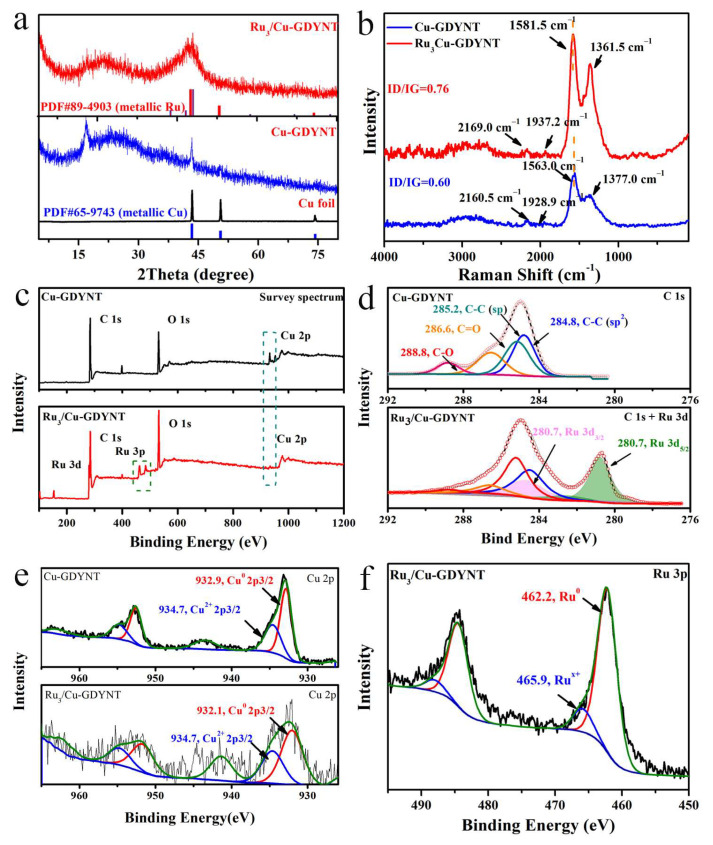
(**a**) The XRD patterns of the Cu foil, Cu-GDYNT, and Ru_3_/Cu-GDYNT; (**b**) Raman spectra of the Cu-GDYNT and Ru_3_/Cu-GDYNT; (**c**) the XPS survey spectra; the high-resolution XPS spectra of (**d**) C 1s, and (**e**) Cu 2p of the Cu-GDYNT and Ru_3_/Cu-GDYNT; (**f**) the high-resolution XPS spectra Ru 3p for Ru_3_/Cu-GDYNT.

Ru_x_/Cu-GDYNT with a different Ru content is prepared in the RuCl_3_ xH_2_O solution without any surfactant using the Cu-GDYNT as the support and reductant, simultaneously. During the reaction, Ru NPs are prepared though the electrodisplacement and reduction reactions by Cu and GDY simultaneously, which is demonstrated by the control experiments below. The X-ray diffraction (XRD) patterns for samples as shown in Figure 3a. The sharp peak of ~16.9° and the wide peak of ~24.5° are derived from GDY. The other diffraction peaks at 43.4°, 50.5°, and 58.3° in the Cu-GDYNT correspond to the (111), (200), and (220) planes of metallic Cu, respectively (PDF# 65-9743), indicating that Cu exists in the form of zero valence element. After the reduction reaction, the other peaks at 38.5°, 42.2°, and 44.1°, can be assigned to the (100), (002), and (101) planes of the hexagonal Ru crystals, demonstrating that Ru_3_/Cu-GDYNT has been successfully synthesized (PDF#06-0663) [33].

In the Raman spectrum of Cu-GDYNT (Figure 3b), the peaks at 1928.9 and 2160.5 cm^−1^ are the stretching vibration peak of the acetylene bond, and the peaks at 1563.0 and 1347.0 cm^−1^ are the G and D bands of GDY, respectively [24]. Compared to that of the Cu-GDYNT, the spectrum of Ru_3_/Cu-GDYNT also shows the same characteristic peak as GDY. However, the G and acetylene bands peaks in the Ru_3_/Cu-GDYNT are blue-shifted by about 18 and 9 cm^−1^, respectively, indicating the presence of strong interaction between Ru and Cu-GDYNT [34]. It is beneficial to anchor the Ru QDs and the regulation of the Ru electronic structure. In addition, the intensity ratio of ID/IG for Ru_3_Cu-GDYNT is 0.76, higher than that of Cu-GDYNT (0.60), which indicates that the Ru_3_Cu-GDYNT structure is much more defective, leading to more active sites for efficient catalysis [35].

The chemical composition of the samples was confirmed using the X-ray photoelectron spectroscopy (XPS) spectrum. The survey spectra show the existence of C, O, and Cu in Cu-GDYNT, and C, O, Cu, and Ru in Ru_3_/Cu-GDYNT. The O is mostly attributable to the oxidation of the terminal alkynyl as well as physically adsorbed and/or trapped oxygen and moisture [16]. Moreover, with the appearance of Ru, the Cu content of the Ru_3_/Cu-GDYNT decreased, indicating the replacement reaction occurred. The C 1s orbital of Cu-GDYNT consists of four subpeaks corresponding to sp^2^ (C=C) at 284.8 eV, sp (C≡C) at 285.2 eV, C=O at 286.6 eV, and C−O at 288.8 eV, respectively [31], demonstrating the existence of sp–sp^2^ co-hybrid structure (Figure 3d) [34]. The ratio of sp–C to sp^2^–C atoms is 1, which is smaller than the ratio of carbon atoms for GDY because Cu–acetylide formed by the combination of some alkyne groups and Cu in GDY obstructs the coupling reaction of the double alkyne bonds [31]. In the spectrum of Ru_3_Cu-GDYNT, the 3d_3/2_ peak at 280.7 eV of Ru^0^ shifts to higher binding energy, compared to the metallic Ru (280.0 eV). The binding energies of Cu in the pristine Cu-GDYNT located at 932.9 and 934.7 eV are ascribed to Cu^0^ 2p_3/2_ and Cu^2+^ 2p_3/2_, respectively. While the corresponding binding energies of Cu^0^ in Ru_3_Cu-GDYNT weaken and shift to lower values compared to that of Cu-GDYNT. These results indicate that Cu-GDYNT, as an electron acceptor, occurs with the electron transfer with Ru, which could effectively modify the electronic configuration of active centers and promote HER kinetics [36]. The Ru 3p high-resolution peaks of Ru_3_Cu-GDYNT are observed in Figure 3f, and the binding energy at 462.2 and 465.9 eV are attributed to the Ru 3p_3/2_ of metallic Ru and Ru^x+^, respectively.

**Figure 4:**
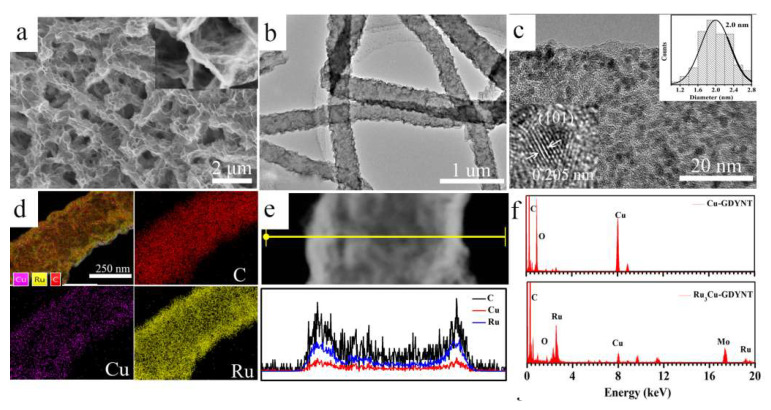
(**a**) The SEM, (**b**) TEM, (**c**) high-resolution TEM images, (**d**) EDS-mapping (**e**) EDS-line scanning images (line scanning depends on atomic content) and (**f**) EDS spectrum of Ru_3_Cu-GDYNT, the inset in (**c**) is statistic diameter of Ru NPs.

The morphology of Ru_3_Cu-GDYNT was also investigated by SEM and TEM. Figure 4a,b show that the morphology of Ru_3_Cu-GDYNT is similar to the Cu-GDYNT, indicating that the structure of the GDYNT will not be changed by the reduction reaction. In the magnified SEM image (the inset in Figure 4a), the dense and dispersed Ru NPs can be seen on the GDY sheets. In the high-resolution TEM image, the NPs are uniformly anchored on the GDY sheet (Figure 4c). The average size distribution of NPs is 2.0 nm summarized in the inset of Figure 4c. With such a narrow particle size distribution range, the Ru NPs are expected to expose a large number of active sites. The crystal lattice spacing of NPs is 0.2 nm attributed to the (101) facets of hexagonal Ru (PDF# 06-0663) (Figure 3a).

EDX mapping images (Figure 4a) and compositional line profiles (Figure 4e) further confirmed the homogeneous anchoring of Ru QDs on Cu-GDYNT. EDS linear scanning shows that the content of Ru in the catalyst is much higher than that of Cu. The ICP test shows the Cu content decrease from 17.7 wt% for Cu-GDYNT to 3.70% for Ru_3_Cu-GDYNT and the Ru content for Ru_3_Cu-GDYNT is 14.8% (Table S3). The above results indicate that most Cu elements were replaced in the catalyst.

**Figure 5:**
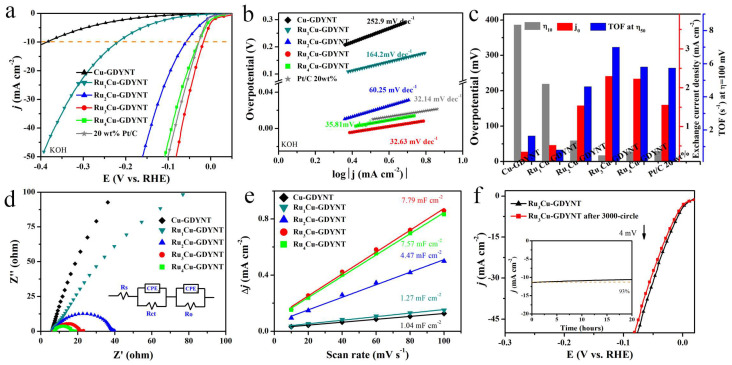
Electrocatalytic HER performance of different catalysts in 1.0 M KOH. (**a**) LSV polarization curves, (**b**) Tafel slopes, (**c**) the comparison of η_10_, the exchange current density (j_0_) and the turnover frequency (TOF) at η = 100 mV; (**d**) Nyquist plots measured at an overpotential of 100 mV, where the inset is equivalent to circuit diagrams; (**e**) The capacitive currents; (**f**) LSVs for 0 and 3000 accelerated durability test cycles, where the inset is a time-dependent current–density curve of Ru_3_Cu-GDYNT under a constant overpotential of 25 mV for 20 h.

The electrochemical HER performance of Ru_x_Cu-GDYNT was first investigated in 1.0 M KOH electrolyte in comparison with Cu-GDYNT, and commercial Pt/C catalysts. According to the inductively coupled plasma-optical emission spectroscopy (ICP-OES) data, the Ru mass loading is 4.83, 11.2, 14.8, and 15.1 wt% for Ru_x_Cu-GDYNT (x = 1, 2, 3, and 4), respectively. As shown in the linear scanning voltammetry (LSV) curves (Figure 5a), Cu-GDYNT without the Ru NPs loading only shows very poor catalytic activity, as reflected by the extremely low current density over the entire potential window. However, Ru_x_Cu-GDYNT exhibits a significantly enhanced HER performance, in terms of both the onset potential and current density. This demonstrates that the Ru on Ru_x_Cu-GDYNT is the real active site for HER. Remarkably, Ru_3_Cu-GDYNT presents the lowest overpotential of merely 17 mV at the current density of 10 mA cm^−2^ among all the samples of Ru_x_Cu-GDYNT (219, 59, and 29 mV to Ru_1_Cu-GDYNT, Ru_2_Cu-GDYNT, and Ru_4_Cu-GDYNT). It is worth noting that the value is even lower than that of the Pt/C sample (η_10_ = 29 mV), revealing superb HER catalytic activities. Moreover, the activity of the Ru_3_Cu-GDYNT catalyst is also superior to those of many other reported Ru-based HER catalysts (Table S1), including Ru and Ni nanoparticles embedded within nitrogen-doped carbon nanofibers (RuNi-NCNFs) (η_10_ = 35 mV, wt_Ru%_ = 28.5%) [37], hybrid ruthenium cobalt phosphide (Ru/CoP) clusters (η_10_ = 56 mV, wt_Ru%_ = 18.4%) [38], and N, P dual-doped carbon-encapsulated ruthenium diphosphide (RuP_2_@NPC) nanoparticle electrocatalyst (η_10_ = 52 mV, wt_Ru%_ = 23.3%) [39].

Subsequently, the HER kinetics of the hybrid catalysts are analyzed as shown in Figure 5b. Ru_3_Cu-GDYNT gives the lowest Tafel slope of 31.21 mV dec^−1^ in the samples of Ru_x_Cu-GDYNT, revealing the most favorable electrocatalytic kinetics on the Ru_3_Cu-GDYNT for the HER. The value of 31.21 mV dec^−1^ indicates that the HER over the catalyst follows the Volmer–Tafel mechanism and the Tafel step is a rate-limiting step. [39] Furthermore, the exchange current density (j_0_) is also calculated based on Tafel plots. The Ru_3_Cu-GDYNT also shows the largest j_0_ of 2.30 mA cm^−2^ (0.35, 0.52, 1.54, 2.23, and 1.56 mA cm^−2^ is for Cu-GDYNT, Ru1Cu-GDYNT, Ru_2_Cu-GDYNT, Ru_4_Cu-GDYNT and Pt/C, respectively), which is about 1.5-fold higher than that of Pt/C. The turnover frequency (TOF) values are calculated in accordance with the surface charges that are proportional to the number of electrochemically active sites (Figure S4) [40]. The number of electrochemically active sites is 2.18 × 10^−9^, 1.25 × 10^−8^, 2.03 × 10^−8^, 4.00 × 10^−8^, 3.02 × 10^−8^, and 3.50 × 10^−8^ mol for Cu-GDYNT, Ru_1_Cu-GDYNT, Ru_2_Cu-GDYNT, Ru_3_Cu-GDYNT, Ru_4_Cu-GDYNT, and Pt/C, respectively. The TOF values are compared at an overpotential of 100 mV in Figure 5c (right and blue). The Ru_3_Cu-GDYNT exhibits the largest amounts of active sites and the highest intrinsic HER activity, which makes Ru_3_Cu-GDYNT rank as the top-tier Pt-free HER electrocatalyst reported to date (Table S1).

Electrochemical impedance spectroscopy (EIS) was employed to investigate the interfacial electron transfer dynamics of the catalyst at the overpotential of 100 mV (Figure 5d). The charge transfer resistance (R_ct_) obtained by fitting is shown in Table S2. Ru_3_Cu-GDYNT exhibits the lowest charge transfer resistance R_ct_ (0.8 Ω), indicating the fastest electron transfer rate during the catalytic reaction [17]. The electrochemical active surface area (ECSA) of each sample was calculated from the electrochemical double-layer capacitance (C_dl_), as this parameter represents a measure of the surface activity of an electrocatalytic material (Figure S5). As illustrated in Figure 5e, the Cdl value of Ru_3_Cu-GDYNT is 7.79 mF cm^−2^, which is larger than the other Ru content catalysts. The minimum R_ct_ and the maximum ECSA of Ru_3_Cu-GDYNT are the intrinsic reasons for the lowest overpotential and the smallest Tafel slope.

Stability is another significant criterion for HER electrocatalysts. As shown in Figure 5f, the HER activities of Ru_3_Cu-GDYNT remain stable even after ADTs for 3000 cycles. The chronopotentiometric curves also suggest that Ru_3_Cu-GDYNT maintain their catalytic activity with negligible decay during long-term electrolysis. After durability testing, the GDYNT still exists and Ru NPs maintain good dispersion on GDYNT, indicating a strong interaction between Cu-GDYNT and Ru QDs (Figure S6).

The catalytic performance of the catalysts in acidic and neutral media is shown in Figure S7 (acidic) and Figure S8 (neutral). The variation of catalytic activity is consistent in alkaline medium, and the sample Ru_3_Cu-GDYNT has the best activity. In 0.5 M H_2_SO_4_ solution, the current density of 10 mA cm^−2^ can be achieved with only 67 mV overpotential and the slope of Tafel is 33.66 mV dec^−1^. In 1.0 M neutral PBS buffer, the required overpotential is 83 mV, and Tafel slope is 60.71 mV dec^−1^. The minimum charge transfer resistance and the maximum C_dl_ of Ru_3_Cu-GDYNT are the reason for its excellent performance (Figures S7d,e and S8d,e). The catalyst can maintain stability over ten hours and 2000 cycles in both acidic and neutral media (Figures S7f and S8f).

### 2.3. The Verification of the Role of Cu

In order to study the role of Cu in the catalyst, the Cu-free GDYNT (Cu_0_-GDYNT) was prepared by washing off the Cu QDs in Cu-GDYNT with 6 M HCl. RuCu_0_-GDYNT was prepared by the same method as Ru_3_Cu-GDYNT using Cu_0_-GDYNT as the support (detail see in ESM). Combined with the XPS result, this proves that the Ru NPs are the result of the electrodisplacement of Cu QDs and the self-reduction for GDY. Figure S9a and S9b show the TEM images of Cu_0_-GDYNT, where part of the wall for Cu_0_-GDYNT is damaged, and there is no Cu QDs on the surface of the GDY nanosheet, indicating that the surface Cu QDs can be removed by concentrated HCl but the structure of the GDYNT is damaged to a certain extent due to the removal of Cu and the corrosion of concentrated acid. The TEM images of Ru/Cu_0_-GDYNT are shown in Figure S9c,d. Dispersed Ru nanoparticles can be observed on the surface of GDY, indicating that Ru is still reduced by GDY without Cu QDs due the low redox potential for the GDY.

**Figure 6:**
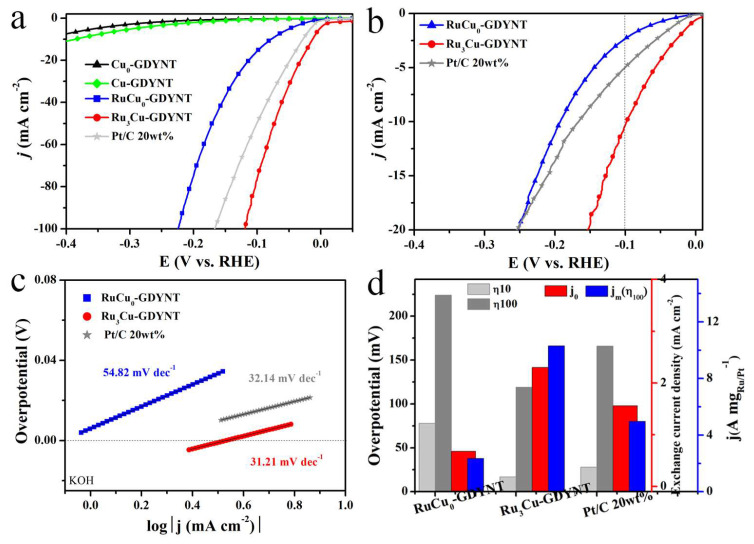
(**a**) The LSV curves, (**b**) mass activity, (**c**) Tafel slope, and (**d**) the comparison for η_10_, η_100_, j_0_, and j_m_ of Cu_0_-GDYNT and Ru/Cu_0_-GDYNT.

The comparisons of the HER performance between Ru/Cu_0_-GDYNT and Ru_3_/Cu-GDYNT are shown in Figure 6. The overpotential to reach the current density of 10 and 100 mA cm^−2^ is 78 and 224 mV, respectively, which is far worse than that of Ru_3_Cu-GDYNT (17 and 119 mV, Figure 6d left). Considering the difference in Ru content, the mass activity of the catalysts is shown in Figure 6b. The mass activity of Ru_3_Cu-GDYNT is the best in the whole test range. When the overpotential is 100 mV, the mass current density of Ru_3_Cu-GDNT is 10.3 A mg_Ru_^−1^, which is 4.4 times of RuCu_0_-GDYNT (2.34 A mg_Ru_^−1^,) and twice of commercial Pt/C (4.97 A mg_Pt_^−1^). Similarly, the Tafel slope and *j*_0_ of RuCu_0_-GDYNT are worse than those of Ru_3_Cu-GDNT. The above experimental results all prove that the presence of Cu in the GDYNT leads to the better performance of the target product.

To gain insight into the origin of the excellent HER performance of Ru_3_Cu-GDYNT, density functional theory (DFT) calculations were further carried out (Figure S10). According to the experimental results, the Ru_55_ cluster model with high stability reported in the literature is used to represent Ru NPs [41]. The majority of Cu QDs on the surface of the GDYNT are replaced by Ru after the galvanic replacement reaction. The content of Cu in Ru_3_Cu-GDYNT and Ru_4_Cu-GDYNT is few changed as measured by ICP (Table S3), indicating that residual Cu has a strong bond cooperation with GDYNT. According to the reports in the literature, the stable existence of a Cu single atom in GDY can represent the Cu-GDYNT model [42].

The differential charge density on the Cu-GDYNT surface shows the electron transfer from the Cu to C surface (0.72 eV). This makes the sp–C of GDY have a higher activity and promotes the binding of the support with Ru (Figure S10a). As well, the binding energy of Ru_55_ to Cu-GDYNT is 7.76 eV, which is higher than that of Ru to GDYNT (7.53 eV) (Figure S10b). The results show that Cu in the GDY plane increased the binding strength between GDY and Ru_55_, which is conducive to the formation of the small-sized Ru QDs, exposing more active sites and improving the stability of the catalyst. The ΔG_H*_ of Ru_55_, Ru_55_/GDYNT, and Ru_55_/Cu-GDYNT are calculated to be 0.077, 0.074, and 0.006 eV, respectively (Figure S10c). At the same time, the number of electron transfer from Ru to C in Ru_55_/GDYNT is 2.24 eV and that in Ru_55_/Cu-GDYNT is 2.55 eV. The results show that the presence of Cu makes the Ru transfer more electrons to C, which weakens the strong adsorption effect of Ru on H and promotes the Tafel step of catalytic reaction, thus obtaining the optimal ΔG_H*_. The optimization of ΔG_H*_ and increased binding energy reveal the reasons for the excellent catalytic activity and stability of Ru_3_Cu-GDYNT. Therefore, as shown in Figure 6d, the coexistence of GDYNT, Ru NPs, and residual Cu makes the catalyst obtain a superior catalytic activity towards HER.

## 3. Materials and Method

### 3.1. Reagents and Materials

Polyacrylonitrile (PAN) was purchased from J&K scientific. Electrodeposited Cu foil was purchased from Kingboard Copper Foil Holding LTD. RuCl_3_ xH_2_O was purchased from Beijing Innochem Science & Technology Co. Beijing, China, Hexakis[(trimethylsilyl)ethynyl]benzene (HEB-TMS) was synthesized by the previously reported reaction [1]. N,N,N’,N’-Tetramethylethylenediamine (TMEDA), and Tetra-nbutylammonium fluoride (TBAF) were purchased from Alfa Esha (China) Chemical Co., LTD. Pyridine, acetone, hydrochloric acid, N, N-dimethylformamide (DMF), ethyl acetate, sodium chloride, and anhydrous sodium sulfate were purchased from Shanghai Aladdin Biochemical Technology Co., LTD. Unless otherwise stated, reagents and solvents were commercially obtained and used without further purification.

### 3.2. Synthesis of the Samples

Synthesis of the PAN nanofibers: 0.4 g of polyacrylonitrile (PAN) was dissolved in 5 mL of DMF solvent with vigorous stirring at 60 °C for 1 h and at room temperature for another 23 h to yield the homogeneously spinning solution. The PAN nanofibers were obtained by the method of electrospinning at 30 °C with a humidity of about 30%. Then, the mixture solution was loaded into a syringe (5 mL) using a stainless-steel nozzle, which was connected to a high-voltage power supply. The parameters of the electrospinning process, including voltage, collecting distance, and propel speed were set at 14 kV, 20 cm, and 0.08 rpm min^−1^, respectively. Fibers were collected on the electrodeposited Cu foil. The resulting fiber was cut into pieces with a size of 3 cm × 4 cm for use.

Synthesis of PAN nanofibers@GDY: HEB monomer was obtained from the desilication reaction of HEB-TMS by TBAF and used immediately. Three PAN nanofibers pieces with the electrodeposited Cu foil were added into a mixed solution of 100 mL acetone, 5 mL pyridine, and 1 mL TMEDA in a three-flask. Either 20 or 50 mg HEB dissolved in 50 mL acetone was slowly added into the aforementioned mixed solution in 4 h. Then, the mixed solution was kept under a nitrogen atmosphere and in a dark place at 50 °C for 12 h. After the reaction, the PAN nanofibers changed from white to dark brown, indicating that GDY was coated on the surface the of PAN nanofibers. Finally, the PAN nanofibers@GDY was washed with acetone to remove the unreacted monomer and dried under nitrogen. During the reaction, the electrolytic Cu foil acts as a substrate for PAN nanofibers, a catalyst for cross-coupling reaction, and a precursor of Cu quantum dots.

The synthesis of the GDY nanotubes (GDYNT): The PAN nanofibers@GDY was etched in DMF at 50 °C to remove the PAN core and residual oligomer. After the reaction, the samples were collected by centrifugation (9800 rpm, 5 min) and washed with the DMF and EtOH several times to remove the residues, and then dried in a vacuum oven. Finally, the as-prepared samples were treated at 180 °C in a tube furnace protected by a mixture of nitrogen-containing 5% hydrogen for 60 min to remove the residues and moisture and improve the metallic Cu content. Since there are abundant Cu quantum dots embedded in the GDY nanotubes, the product is denoted as Cu-GDYNT.

The synthesis of the Ru_x_Cu-GDYNT: Ru_x_Cu-GDYNT was obtained by the galvanic replacement process. In detail, 10 mg of the prepared powder was sonically dispersed in 5 mL of 1,3-Propanediol for 10 min to obtain a homogeneous dispersion of Cu-GDYNT. Then, a certain volume X (X = 100, 200, 300, and 400 μL) of 0.1 M RuCl_3_ xH_2_O solution was added to the above dispersion under the protection of nitrogen and kept in an oil bath at 160 °C for 12 h under stirring. The resulting samples were washed with DI water, collected by filtration, and dried in a vacuum oven, denoted as Ru_1_Cu-GDYNT, Ru_2_Cu-GDYNT, Ru_3_Cu-GDYNT, and Ru_4_Cu-GDYNT, respectively.

Synthesis of the Cu_0_-GDYNT and RuCu_0_-GDYNT: Cu_0_-GDYNT represents the sample of GDYNT without Cu, and comes from Cu-GDYNT treated with 6 M HCl over 12 h. The Ru-GDYNT was obtained by the same procedure, only substituting Cu_0_-GDYNT for Cu-GDYNT.

### 3.3. Characterization

The morphology and structure of nanomaterials were observed via field emission scanning electron microscopy (FESEM, Hitachi S-4800) and transmission electron microscopy (TEM JEOL JEM-2100F) with an acceleration voltage of 200 kV. The EDS line-scan and HAADF-STEM elemental mapping analysis were also performed using JEM 2100F equipped with an Oxford Instrument energy dispersive spectrometer. The X-ray diffraction (XRD) was recorded on an X-ray diffractometer (XRD, SmartLab, using filtered Cu Kα radiation). The surface characteristics of materials were tested by X-ray photoelectron spectroscopy (XPS) carried out on an ESCALAB 250 spectrophotometer with Al-Kα radiation. Raman spectra were performed by Bruker Senterra Raman spectrometer with a laser excitation wavelength of 532 nm.

### 3.4. Electrochemical Measurements

Electrolysis experiments were carried out using a typical three-electrode system with an H-style cell on an electrochemical working station (CHI 760E, Shanghai CH Instruments, China). The 1.0 M KOH, 0.5 M H_2_SO_4_, and 1.0 M PBS (pH = 7) solution saturated with Ar served as the alkaline, acidic, and neutral electrolyte for the HER activity test. The acidic and neutral electrochemical measurements were performed using an Ag/AgCl with a saturated KCl electrode as the reference electrode, while the alkaline electrochemical measurements were performed using a calomel electrode (SCE) as the reference electrode. The graphite plate was used as the counter electrode in all measurements.

The working electrode was prepared as follows: 5 mg of each freshly prepared catalyst (or commercial Pt/C, 20 wt%, Alfa Aesar), 0.4 mg of acetylene black (conductive agent), and 20 µL of 5 wt% Nafion solution (Sigma-Aldrich) were dispersed in 980 µL of a water/anhydrous ethanol mixed solvent (1:1 volume ratio) by 1 h of sonication to form a homogeneous ink. Then, 10 µL of the catalyst ink was dropped onto the surface of a polished GC electrode (L-type) (GCE, 4.0 mm diameter, 0.1265 cm^−2^) and dried in air. The loading amount was calculated to be 0.4 mg cm^−2^.

For HER measurements, cyclic voltammograms (CVs) were conducted with a scan rate of 100 mV s^−1^ as the activation process. All the used polarization curves are the steady-state ones after several cycles. The HER polarization curves were acquired by linear sweep voltammetry (LSV) at a scan rate of 5 mV s^−1^. In all measurements, the reference electrode was calibrated concerning the reversible hydrogen electrode (RHE). All polarization curves were iR-corrected. Electrochemical impedance spectroscopy (EIS) was carried out from 10^5^ to 10^−1^ Hz, with an AC amplitude of 5 mV under the same potential. The iR-corrected potential was obtained after the correction of internal resistance measured by EIS following the equation:

E_(RHE)_ = E_0_ + E_SCE_ or _Ag/AgCl_ − I × R_s_ + 0.059 × pH.

The electrochemically active surface area (ECSA) of the samples was estimated using a simple cyclic voltammetry (CV) method. The electrochemical double-layer capacitance (C_dl_) is from linear fitting, and the slope is the C_dl_ value.

## 4. Conclusions

In conclusion, the GDYNT with a controllable diameter is successfully fabricated using electrospun PAN fibers as templates. The in situ formed Cu QDs with the size of 2.2 nm are uniformly embedded in the GDYNT. Using Cu-GDYNT as the support, the Ru NPs supported by Cu-modified GDYNT are produced. Experimental results and theoretical calculations show that there is a strong electronic interaction between Ru and the support Cu-GDYNT and the existence of Cu in the GDYNT optimizes the electron transfer from Ru to GDYNT. The prepared catalyst shows an excellent HER catalytic performance in the full pH range, requiring 17, 67, and 83 mV in 1.0 M KOH, 0.5 M H_2_SO_4_, and 1.0 M neutral PBS solutions, respectively. This work provides a new method for the controllable preparation of GDYNT, and such GDYNT can be applied in catalysis and other sustainable energy applications.

## Data Availability

Data are contained within the article and Supplementary Materials.

## Supplementary Materials

The following supporting information can be downloaded at: https://www.mdpi.com/article/10.3390/molecules29061410/s1. References [43,44,45,46,47,48,49,50,51,52,53,54,55,56,57,58,59] are citation in the Supplementary Materials.
