# Supplementary material for: Preparation and Support Effect of Graphdiyne Nanotubes with Abundant Cu Quantum Dots
Source: Molecules. 2024 Mar 21;29(6):1410. doi: 10.3390/molecules29061410 (PMC10974350; doi:10.3390/molecules29061410)
Supplement: Supplementary file 1 [file molecules-29-01410-s001.zip › molecules-2903613-supplementary.pdf]

Supporting Information

Preparation and Support Effect of Graphdiyne Nanotubes with Abundant Cu Quantum Dots

Yan Lv *,†, Wenzhou Wang †, Zhangwei Li and Fucang Liang

State Key Laboratory of Chemistry and Utilization of Carbon Based Energy Resources, College of Chemistry, Xinjiang University, Urumqi 830017, China

* Correspondence: lvyan2014@xju.edu.cn

† These authors contributed equally to this work.

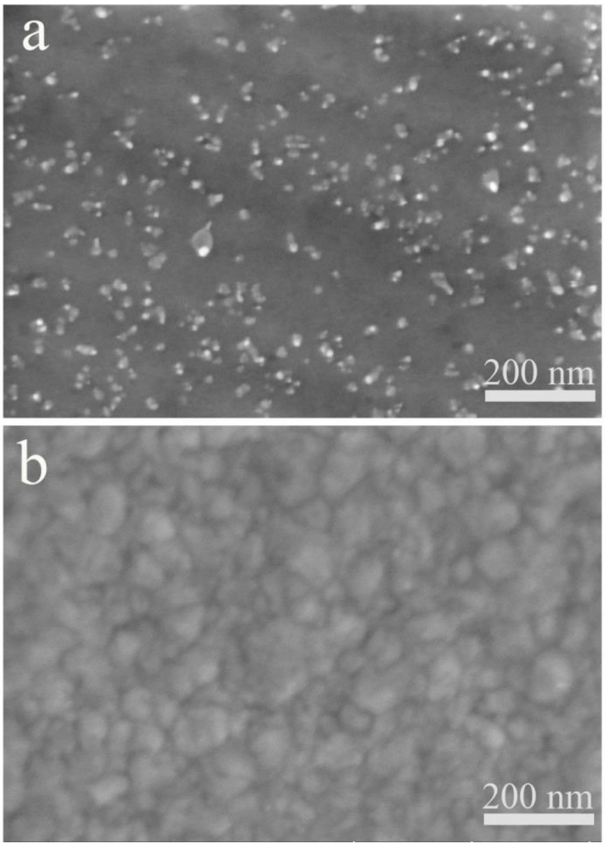

Figure S1. Microstructure of the (a) general Cu foils (Sinopharm Chemical Reagent Co. Ltd. Used as a catalyst for the synthesis of most GDY), and (b) electrodeposited Cu foil.

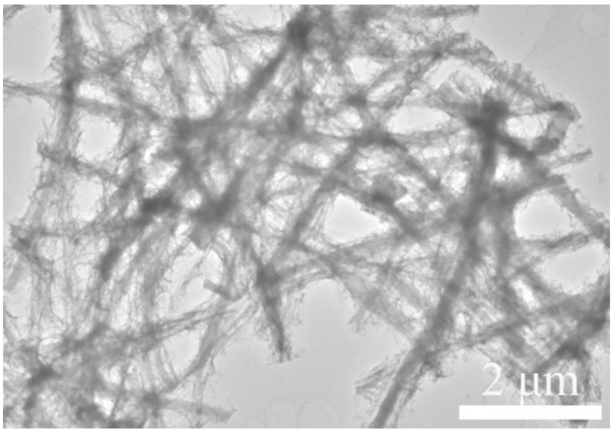

Figure S2. The prepared GDYNT with an inner diameter of 80 nm (The PAN nanofibers using the stainless steel needle number of NO. 20).

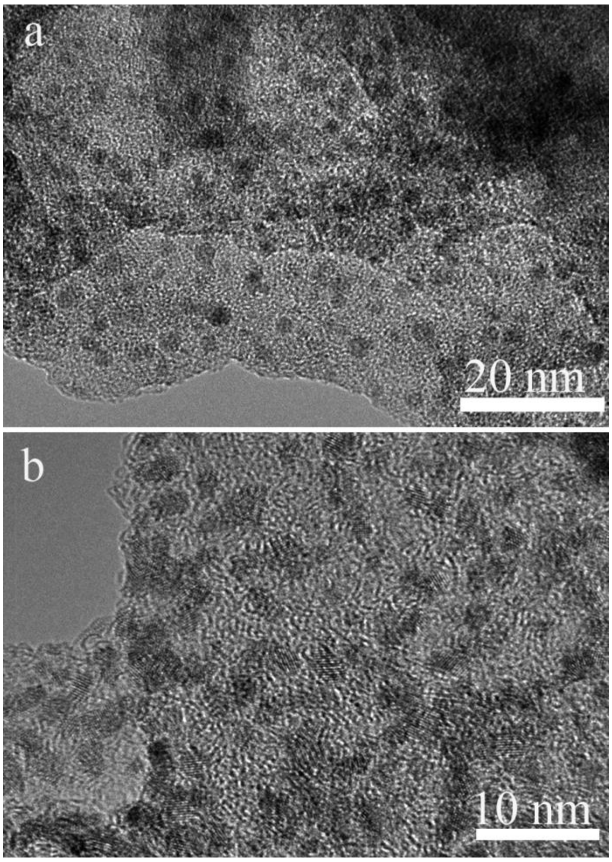

Figure S3. (a,b)The high-resolution TEM images of Cu-GDYNT in different positions.

Active site calculation: The electrode was prepared as mentioned above. CV measurements were carried out in PBS electrolyte (pH=7) [1]. Then, the absolute components of the voltammetric charges (cathodic and anodic) reported during one single blank measurement were added. Assuming a one-electron redox process, this absolute charge was divided by two. The value was then divided by the Faraday constant to get the number of active sites (n), that is n= Q/2F.

TOF calculation： The turnover frequency (s^{-1}) was calculated following equation:

TOF = I/2nF

I: Current (A) during the LSV measurement in 1.0 M KOH.

F: Faraday constant (C mol^{-1}).

n: Number of active sites (mol).

The factor 1/2 arrives by taking into account that two electrons are required to form one hydrogen molecule from two protons.

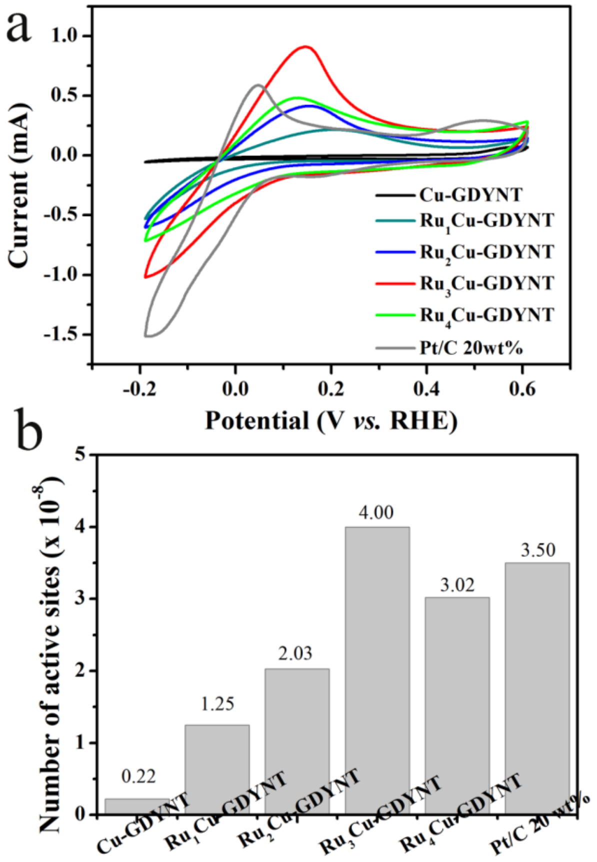

Figure S4. (a) Cyclic Voltammograms of Cu-GDYNT, Ru_xCu-GDYNT, and Pt/C (20 wt % Pt) catalysts measured in PBS solution (pH = 7) at 50 mV s^{-1}; (b) the number of active sites of samples.

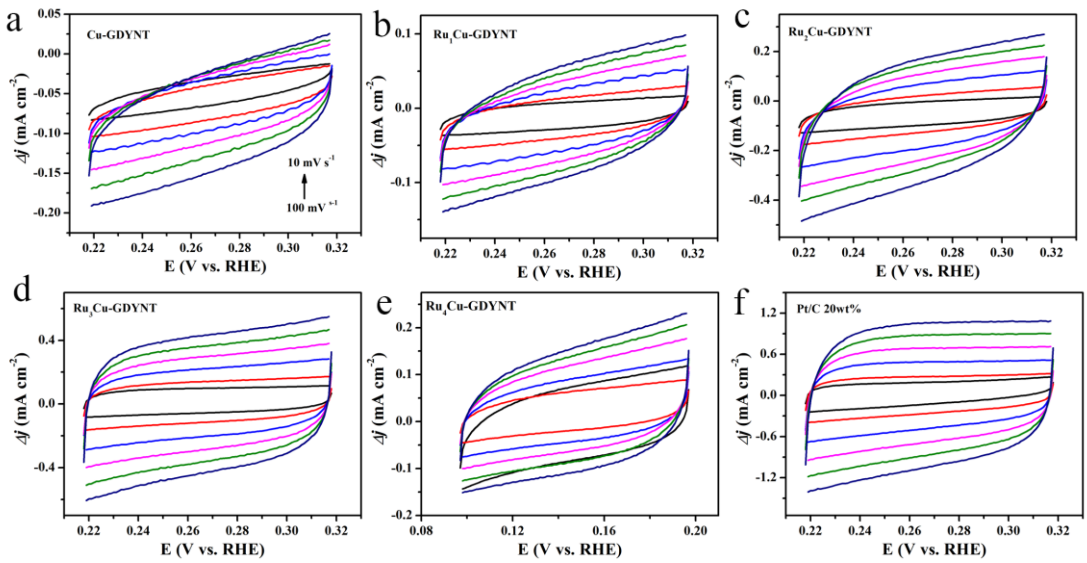

Figure S5. CV curves of the (a) Cu-GDYNT, (b) Ru_1Cu-GDYNT, (c) Ru_2Cu-GDYNT, (d) Ru_3Cu-GDYNT, (e) Ru_4Cu-GDYNT, (f) Pt/C20wt% at various scan rates to investigate the specific capacitor values (C_{dl}) that were used to estimate the electrochemical active specific surface area for HER.

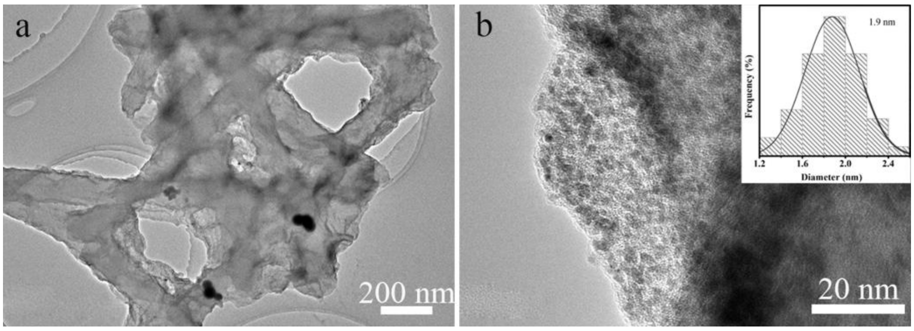

Figure S6. (a) TEM image and (b) the High-resolution TEM image of Ru_3Cu-GDYNT after stability test in 1.0 M KOH solutions. the inset in (b) is statistic diameter of Ru NPs.

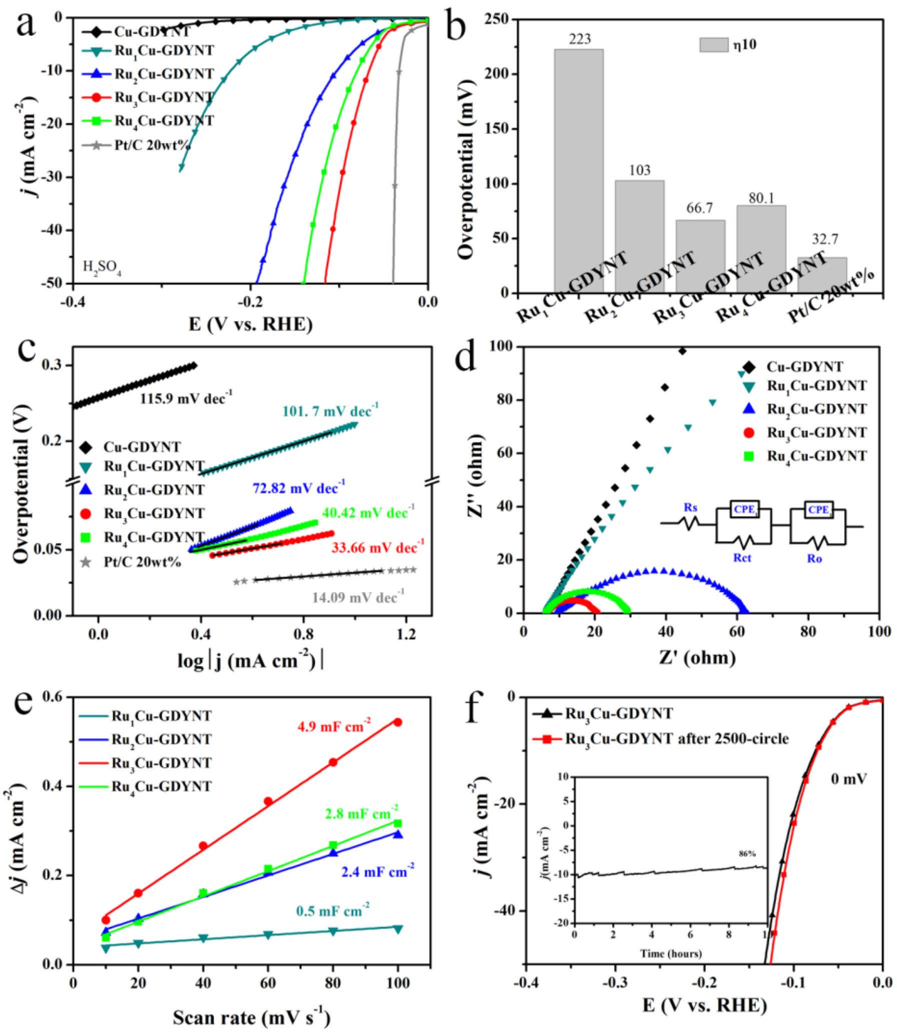

Figure S7. Electrocatalytic HER performance of different catalysts in 0.5 M H_2SO_4 (a) LSV polarization curves, (b) the comparison of η_{10}, (c) the Tafel slopes, (d) Nyquist plots measured at an overpotential of 100 mV, the inset is equivalent circuit diagrams, (e) the capacitive currents, (f) LSVs for 0 and 2500 accelerated durability test cycles, the inset is time-dependent current density curve of Ru_3Cu-GDYNT at the current density of 10 mA cm^{-2} for 20 h.

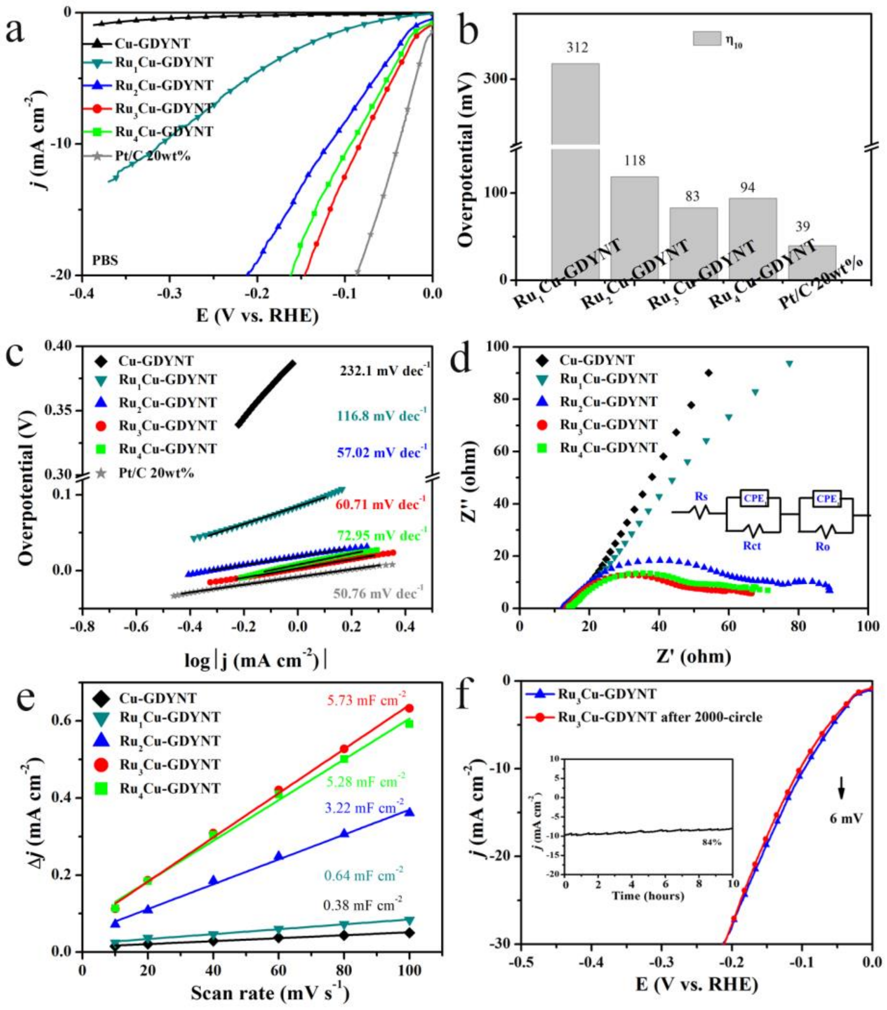

Figure S8. Electrocatalytic HER performance of different catalysts in 1.0 M PBS (pH=7.0). (a) LSV polarization curves, (b) the comparison of η_{10}, (c) the Tafel slopes, (d) Nyquist plots measured at an overpotential of 100 mV, the inset is equivalent circuit diagrams, (e) the capacitive currents, (f) LSVs for 0 and 2000 accelerated durability test cycles, the inset is time-dependent current density curve of Ru_3Cu-GDYNT at the current density of 10 mA cm^{-2} for 10 h.

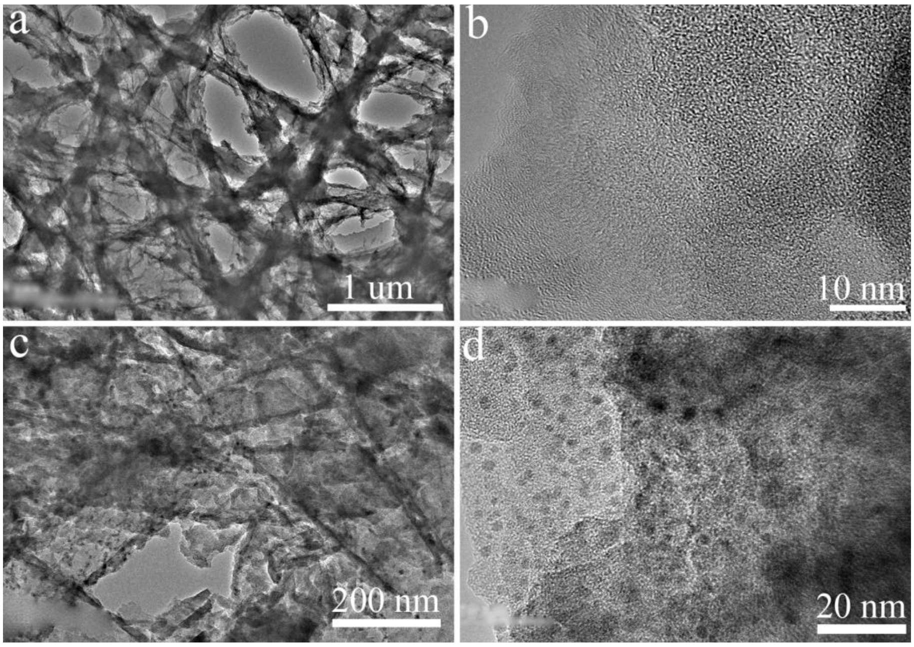

Figure S9. The TEM images of (a, b) Cu_0-GDYNT, and (c, d) $RuCu_0$-GDYNT.

DFT Calculations: The Vienna ab-initio simulation package (VASP) based on density functional theory (DFT) was employed to carry out first-principles calculations [2]. In the calculation of structure relaxation, exchange and correlation effects were considered using Perdew-Burke-Ernzerhof functional (GGA-PBE)[3,4]. The long-range van der Waals (vdW) interactions were corrected for dispersion interaction by using the zero damping DFT-D3 method of Grimme. The plane wave energy cutoff was set as 500 eV. The force convergence of 0.02 eV·Å^{-1}, the self-consistency accuracy of 10^{-4} eV.

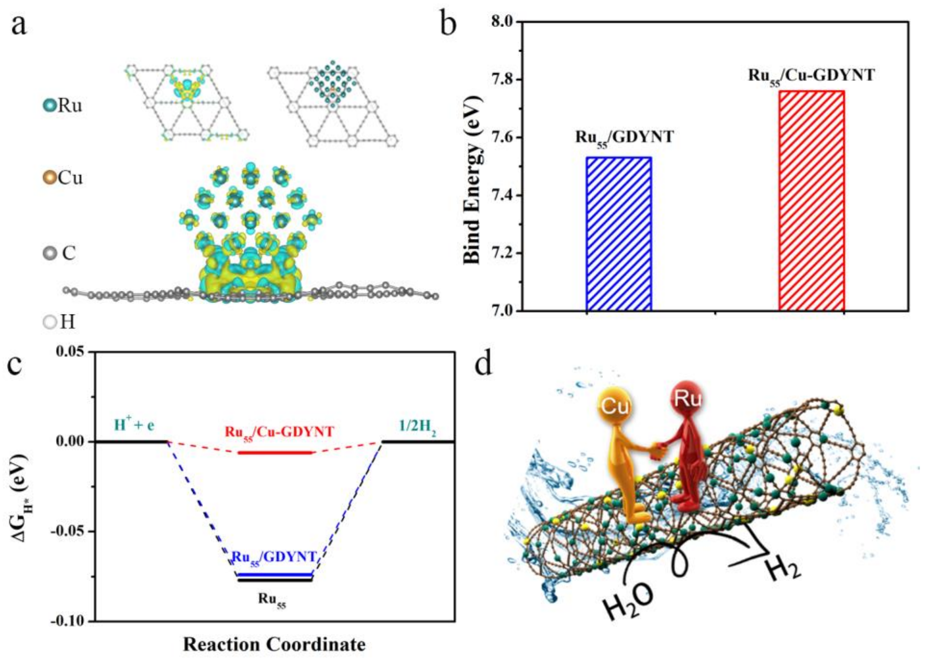

Figure S10. (a) The charge density distribution images of Cu-GDYNT and Ru_{55}/Cu-GDYNT, (b) the binding energies of Ru_{55} with GDYNT and Cu-GDYNT, (c) calculated ΔG_{H*}, (d) schematic diagram of catalyst performance contribution.

Table S1. Comparison of the η_{10}, Tafel slope, and Ru content of Ru_3Cu/GDYNT with other Ru-based catalysts in 1.0 M KOH

**Electrocatalysts**	**η_{10} (mV)**	**Tafel slope/mA dec^{-1}**	**Ru wt%**	**Ref.**
**Ru_3Cu-GDYNT**	**17.8**	**32.63**	**14.7**	**This Work**
**Pt/C**	**28.9**	**32.14**	**20**	**This Work**
Ru/NiFe LDH-F/NF	115.6	87.1	0.43	5
Ru_1Co_2 NPs	188	66.5	-	6
Ru_1Ni_1-NCNFs	35	30	28.2	7
Ru/CoP	56	37	18.4	8
RuCoP	23	37	17.7	8
Ru/C_3N_4/C	79	-	20	9
Ru–Ru_2P/PC	49	35.1	14.9	10
RuP_2@NPC	52	38	23.3	11
RuP_x@NPC	74	70	-	12
Ru_{SA+NP}/DC	18.8	35.8	68.6	13
Ru/NHCSs	26	54.1	-	14
Ru-RuO_2/CNT	12	64	57.6	15
Ru@GnP	22	28	10.7	16
$Cu_{2-x}S$@Ru NPs	82	48	20	17
(Ru-Co)O_x	44.1	23.5	28.4	18
Ru_2Ni_2SNs/C,	40.0	23.4	5.5	19
NiRu@N-C	32	64	1.862	20
Ni@Ni_2P−Ru	31	41	-	21

Table S2. Fitting values of the electrochemical impedance of samples in solutions of different pH values

R_{ct}（Ω）	Cu-GDYNT	Ru_1Cu-GDYNT	Ru_2Cu-GDYNT	Ru_3Cu-GDYNT	Ru_4Cu-GDYNT
1.0 M KOH	714.6	343.5	1.540	0.8051	4.424
0.5 M H_2SO_4	12580	584.2	3.928	0.4648	0.6644
1.0 MPBS	4647	319.8	32.13	19.37	23.24

Table S3. Metal content in the sample measured by ICP-AES.

wt%	Cu-GDYNT	Ru_1Cu-GDYNT	Ru_2Cu-GDYNT	Ru_3Cu-GDYNT	Ru_4Cu-GDYNT
Cu	17.7	10.1	4.82	3.70	3.51
Ru	0	4.85	11.2	14.8	15.1

Reference:

[1] Tian J, Liu Q, Asiri A M, et al. Self-supported nanoporous cobalt phosphide nanowire arrays: an efficient 3D hydrogen-evolving cathode over the wide range of pH 0-14. Journal of the American Chemical Society, 2014, 136(21): 7587-7590.

[2] Kresse G, Furthmüller J. Efficiency of ab-initio total energy calculations for metals and semiconductors using a plane-wave basis set. Computufional Murerials Science, 1996, 6: 15-50.

[3] Blöchl P E. Projector augmented-wave method. Physical Review B, 1994, 50(24): 953-978.

[4] Perdew J P, Chevary J A, Vosko S H, et al. Atoms, molecules, solids, and surfaces: Applications of the generalized gradient approximation for exchange and correlation. Annual Review of Condensed Matter Physics, 1992, 46(11): 6671-6687.

[5] Wang Y, Zheng P, Li M, et al. Interfacial synergy between dispersed Ru sub-nanoclusters and porous NiFe layered double hydroxide on accelerated overall water splitting by intermediate modulation. Nanoscale, 2020, 12(17): 9669-9679.

[6] Bao Y, Dai J, Zhao J, et al. Modulation in ruthenium-cobalt electronic structure for highly efficient overall water splitting. ACS Applied Energy Materials, 2020, 3(2): 1869-1874.

[7] Li M, Wang H, Zhu W, et al. RuNi nanoparticles embedded in N-doped carbon nanofibers as a robust bifunctional catalyst for efficient overall water splitting. Advanced Science, 2020, 7(2): 1901833

[8] Xu J, Liu T, Li J, et al. Boosting the hydrogen evolution performance of ruthenium clusters through synergistic coupling with cobalt phosphide. Energy & Environmental Science, 2018, 11(7): 1819-1827.

[9] Zheng Y, Jiao Y, Zhu Y, et al. High electrocatalytic hydrogen evolution activity of an anomalous ruthenium catalyst[J]. Journal of the American Chemical Society, 2016, 138(49): 16174-16181.

[10] Liu Z, Li Z, Li J, et al. Engineering of Ru/Ru_2P interfaces superior to Pt active sites for catalysis of the alkaline hydrogen evolution reaction. Journal of Materials Chemistry A, 2019, 7(10): 5621-5625.

[11] Pu Z, Amiinu I S, Kou Z, et al. RuP2-based catalysts with platinum-like activity and higher durability for the hydrogen evolution reaction at all pH values. Angewandte Chemie International Edition, 2017, 56(38): 11559-11564.

[12] Chi J Q, Gao W K, Lin J H, et al. Hydrogen evolution activity of ruthenium phosphides encapsulated in nitrogen- and phosphorous-codoped hollow carbon nanospheres. ChemSusChem, 2018, 11(4): 743-752.

[13] Zhang L, Jang H, Wang Y, et al. Exploring the dominant role of atomic-and nano-ruthenium as active sites for hydrogen evolution reaction in both acidic and alkaline media. Advanced Science, 2021, 8(15): 2004516.

[14] Ding R, Yan T, Wang Y, et al. Carbon nanopore and anchoring site-assisted general construction of encapsulated metal (Rh, Ru, Ir) nanoclusters for highly efficient hydrogen evolution in pH-universal electrolytes and natural seawater. Green Chemistry, 2021, 23(12): 4551-4559.

[15] Zhang M, Chen J, Li H, et al. Ru-RuO_2/CNT hybrids as high-activity pH-universal electrocatalysts for water splitting within 0.73 V in an asymmetric-electrolyte electrolyzer. Nano Energy, 2019, 61: 576-583.

[16] Li F, Han G F, Noh H J, et al. Mechanochemically assisted synthesis of a Ru catalyst for hydrogen evolution with performance superior to Pt in both acidic and alkaline media. Advanced Materials, 2018, 30(44): 1803676.

[17] Yoon D, Lee J, Seo B, et al. Cactus-like hollow $Cu_{2-x}S$@Ru nanoplates as excellent and robust electrocatalysts for the alkaline hydrogen evolution reaction. Small, 2017, 13(29): 1700052

[18] Wang C, Qi L. Heterostructured inter-doped ruthenium-cobalt oxide hollow nanosheet arrays for highly efficient overall water splitting. Angewandte Chemie International Edition, 2020, 59(39): 17219-17224.

[19] Ding J, Shao Q, Feng Y, et al. Ruthenium-nickel sandwiched nanoplates for efficient water splitting electrocatalysis. Nano Energy, 2018, 47: 1-7.

[20] Xu Y, Yin S, Li C, et al. Low-ruthenium-content NiRu nanoalloys encapsulated in nitrogen-doped carbon as highly efficient and pH-universal electrocatalysts for the hydrogen evolution reaction. Journal of Materials Chemistry A, 2018, 6(4): 1376-1381.

[21] Liu Y, Liu S, Wang Y, et al. Ru modulation effects in the synthesis of unique rod-like Ni@Ni_2P-Ru heterostructures and their remarkable electrocatalytic hydrogen evolution performance. Journal of the American Chemical Society, 2018, 140(8): 2731-273.
